# Loneliness and Sleep Quality Among Older Adults Living in Nursing Homes

**DOI:** 10.3390/nursrep16050173

**Published:** 2026-05-19

**Authors:** Rui Novais, Cláudia Rodrigues, Fátima Braga, Rui Pereira, Carlos Sequeira, Núria Albacar-Riobóo, Silvana Martins, Odete Araújo

**Affiliations:** 1 School of Nursing, University of Minho, 4710-057 Braga, Portugal; rnovais@ese.uminho.pt (R.N.); fbraga@ese.uminho.pt (F.B.); ruipereira@ese.uminho.pt (R.P.); 2 Nursing Research Centre, University of Minho, 4710-057 Braga, Portugal; claudiailrodrigues15@gmail.com; 3 Faculty of Nursing, University of Rovira iVirgili (URV), 43003 Tarragona, Spain; nuria.albacar@urv.cat; 4 Health Sciences Research Unit: Nursing (UICISA: E), Nursing School of Coimbra (ESEnfC), 3045-043 Coimbra, Portugal; 5 School of Nursing, University of Porto, 4200-072 Porto, Portugal; carlossequeira@esenf.pt; 6 RISE-Health, Nursing School of Porto, 4200-450 Porto, Portugal; 7 ProChild CoLAB Against Poverty and Social Exclusion—Association, 4810-225 Guimarães, Portugal; silvana.martins@prochildcolab.pt

**Keywords:** ageing, nursing homes, loneliness, sleep quality, institutionalised older adults

## Abstract

**Background:** Population ageing has increased the number of older adults living in nursing homes, where loneliness and sleep disturbances are prevalent and negatively affect well-being. Evidence suggests a bidirectional relationship between loneliness and sleep quality, although research in institutionalised populations remains limited. **Objectives:** This study aimed to characterise the sociodemographic and health profile of nursing home residents in Northern Portugal and examine associations between sleep quality, loneliness, sociodemographic and health variables. **Methods:** A cross-sectional study was conducted with 157 older adults (≥65 years) across 13 nursing homes. Data were collected using a sociodemographic questionnaire and the Portuguese version of UCLA Loneliness Scale, Pittsburgh Sleep Quality Index and Montreal Cognitive Assessment. Pearson correlations and hierarchical multiple regression analyses were performed. **Results:** Participants were predominantly female (72.6%), widowed (55.4%), and aged ≥80 years. Most reported chronic conditions (98.7%) and limitations in activities of daily living (75.2%). Age showed modest positive correlations with loneliness. Loneliness dimensions were strongly associated with poorer sleep quality and greater daytime dysfunction. Hierarchical regression revealed that sociodemographic variables explained only a small proportion of variance in sleep quality. The addition of loneliness variables increased explained variance to 38.1%, highlighting loneliness as a key psychosocial predictor. **Conclusions:** Loneliness significantly influences sleep quality among older adults living in nursing homes. Interventions should integrate strategies to enhance social engagement alongside sleep hygiene measures. Longitudinal studies are recommended to clarify causal pathways.

## 1. Introduction

Population ageing represents one of the most significant demographic transformations shaping contemporary societies. In Europe, this transition has been particularly pronounced, with individuals aged 65 years and over representing 21.6% of the population of the European Union in 2024. This proportion is projected to increase substantially in the coming decades, potentially reaching 32.5% by 2100 [1]. This demographic shift is largely driven by sustained declines in fertility alongside increasing life expectancy, resulting in a progressive restructuring of the population age distribution and a growing proportion of older adults.

Portugal is among the countries most affected by this demographic transformation. Older adults currently account for 24.1% of the Portuguese population, placing the country among those with the highest levels of population ageing both within the EU and globally [1]. Moreover, the older population has been growing at an annual rate exceeding 2% since 2019, reflecting the accelerated pace of demographic ageing in the national context [2]. These trends pose important challenges for health systems and long-term care structures, particularly regarding the provision of adequate support for older adults experiencing increasing levels of dependency and complex health needs.

Ageing is widely recognised as a multidimensional process shaped by the interaction of biological, psychological, and social factors across the life course [3]. Ageing trajectories are therefore highly heterogeneous, reflecting differences in genetic predispositions, life experiences, social environments, lifestyle behaviours, and access to healthcare resources [4]. As individuals age, the cumulative effects of chronic conditions, functional limitations, and frailty may progressively compromise independence and overall quality of life.

Although most older adults express a preference to remain in their own homes—a concept commonly referred to as ageing in place—this preference is not always feasible in practice [5,6]. The progression of chronic diseases, together with declines in physical and cognitive functioning, frequently leads to reduced autonomy in both basic and instrumental activities of daily living [7]. In Portugal, approximately 60% of individuals aged 65 years and over report experiencing some degree of functional limitation in their daily lives [8]. In such circumstances, institutionalisation may become a necessary alternative when home-based care is no longer viable. Decisions regarding institutionalisation are often influenced by multiple factors, including deteriorating health, cognitive impairment, advanced age, and the absence of adequate informal support networks [5,9,10].

Consequently, nursing homes have become an increasingly important component of the long-term care system in Portugal. Approximately 3.8% of older adults currently live in nursing homes, distributed across more than 2500 facilities nationwide [11,12]. The sustained demand for institutional care is reflected in high occupancy rates, which reached 91.8% of available places in mainland Portugal in 2022. Although these institutions primarily aim to address older adults’ physical and functional care needs, increasing attention has been directed towards the psychosocial and mental health challenges experienced by this population.

Among these challenges, loneliness has emerged as a particularly salient concern among institutionalised older adults. Loneliness is commonly defined as the subjective perception of a discrepancy between desired and actual social relationships [13,14] and is increasingly recognised as an important determinant of health and well-being in later life [15,16]. Older adults living in nursing homes may be especially vulnerable to loneliness due to disruptions in established social networks, relocation from familiar environments, and reduced opportunities for meaningful social interaction [17,18]. Empirical evidence indicates that a substantial proportion of institutionalised older adults experience loneliness, with approximately 61% reporting moderate levels and around 35% severe levels [19]. Loneliness in later life has also been associated with poorer psychological well-being, increased depressive symptoms, reduced quality of life, and higher mortality risk [20,21,22].

Sleep disturbances are also highly prevalent among older adults and appear to be particularly common in institutional care settings. Ageing is associated with physiological changes in sleep architecture, including reduced slow-wave sleep, increased nocturnal awakenings, and greater sleep fragmentation [23,24,25]. In addition to their high prevalence, sleep disturbances in older adults also exhibit gender differences, with evidence indicating variations in both the frequency and nature of sleep complaints between men and women [25]. Environmental conditions frequently present in nursing homes—such as noise, nocturnal care routines, shared rooms, and limited exposure to natural light—may further contribute to sleep disruption [26,27]. Consequently, more than half of older adults living in nursing homes are estimated to experience at least one chronic sleep disorder [28]. Poor sleep quality in later life has been consistently associated with adverse health outcomes, including cognitive decline, depressive symptoms, cardiovascular disease, functional impairment, and increased mortality risk [29,30,31].

Increasing attention has therefore been directed towards the relationship between loneliness and sleep quality in older adults. A growing body of research suggests that these phenomena are closely interconnected and may influence each other through multiple psychological and physiological mechanisms [14,32]. From a psychological perspective, loneliness may heighten perceptions of vulnerability and social threat, leading to increased cognitive arousal and hypervigilance that interfere with the ability to initiate and maintain sleep [14,33]. Physiologically, loneliness has been associated with dysregulation of neuroendocrine responses, increased cortisol levels, and impaired immune functioning, all of which may disrupt normal sleep regulation processes [34,35].

Importantly, the relationship between loneliness and sleep appears to be bidirectional. However, given the cross-sectional nature of most studies in institutional settings, including the present one, the current analysis focuses on identifying factors associated with sleep quality, without inferring causal direction. While loneliness may contribute to poorer sleep quality, sleep disturbances may also exacerbate feelings of social disconnection and emotional deprivation, thereby reinforcing experiences of loneliness [36,37]. Longitudinal evidence supports this reciprocal relationship. For instance, Hawkley et al. [14] found that loneliness predicted subsequent sleep fragmentation even after controlling for depressive symptoms and other health conditions. Similarly, Jiang et al. [38] demonstrated that social isolation and sleep are closely linked in older adults, with loneliness acting as a mediating mechanism between perceived social connectedness and sleep outcomes. More recently, Liu et al. [39] reported that loneliness significantly increases the risk of depressive symptoms both directly and indirectly through the deterioration of sleep quality.

Despite increasing international attention to the relationship between loneliness and sleep in later life, several important gaps remain in the literature. Most studies have focused on community-dwelling older adults, while comparatively fewer have examined these relationships in institutional settings, where both loneliness and sleep disturbances may be particularly pronounced. Furthermore, the psychosocial determinants of sleep among older adults living in nursing homes remain insufficiently explored. Evidence from Southern European countries is also limited, particularly in Portugal, where the mental health profile of institutionalised older adults remains poorly documented.

Sleep quality in older adults can be conceptualised within a biopsychosocial framework in which biological, psychological, and social determinants interact to influence sleep regulation. Ageing-related biological changes, including alterations in circadian rhythm stability, sleep architecture fragmentation, and increased burden of chronic comorbidities, contribute to diminished sleep continuity and efficiency. Within the psychological domain, loneliness has been identified as a salient risk factor for impaired sleep, acting through heightened cognitive and affective arousal, including increased rumination, perceived social threat, and hypervigilance, which disrupt sleep initiation and maintenance. Depressive and anxiety symptoms may further amplify this effect by reinforcing maladaptive cognitive–emotional processes. At the social level, reduced social integration, social isolation, and institutional living conditions increase exposure to loneliness and limit access to perceived and actual social support. Accordingly, loneliness may function as a key mediating mechanism linking adverse social conditions to poorer sleep outcomes in later life [24].

Addressing these gaps is essential for improving understanding of the psychosocial determinants of well-being in institutionalised populations and for informing interventions aimed at promoting mental health and quality of life in nursing homes.

Therefore, the present study aims to investigate the association between sleep quality and loneliness among older adults living in nursing homes in Northern Portugal. Specifically, the objectives of this study are: (i) to describe the sociodemographic and health profile of institutionalised older adults in this region; and (ii) to explore the associations between sleep quality, loneliness, and the sociodemographic and health characteristics of this population. Although bidirectional associations have been reported, this study adopts sleep quality as the outcome variable to examine psychosocial correlates in an institutionalised context.

## 2. Materials and Methods

### 2.1. Study Design

This correlational, descriptive study within a cross-sectional design was conducted in 13 nursing homes located in Northern Portugal.

This study was guided by a conceptual framework in which sleep quality is understood as a multidimensional outcome influenced by demographic, institutional, and psychosocial factors. Accordingly, global sleep quality and its underlying components were examined in relation to age, institutional context, social support, and loneliness-related dimensions.

### 2.2. Context and Setting

Nursing homes are specialised facilities that provide multidisciplinary health and social care to older adults who live there permanently and present significant healthcare needs or insufficient social support.

The participating nursing homes varied in size, accommodating between 29 and 198 older adults. Staffing structures also differed as all facilities employed healthcare assistants, while nursing coverage ranged from 24 h availability to daytime-only presence. In addition, some nursing homes had periodic support from other healthcare professionals, such as physicians, psychologists, and physical therapists.

### 2.3. Population and Sampling

Older adults were recruited from several nursing homes using a non-probability convenience sampling technique, based on the accessibility and availability of members of the target population. This methodological option was justified by the time, resource, and logistical constraints inherent to the data collection process.

The final sample comprised 157 individuals who met the inclusion criteria, namely aged 65 years or older, living in a nursing home, and exhibiting an initial or intermediate stage of cognitive impairment. According to the study protocol, individuals with severe cognitive impairment or who were unable to communicate verbally were excluded.

### 2.4. Measures

#### 2.4.1. Sociodemographic Questionnaire

A sociodemographic questionnaire was developed specifically for the purpose of this project. It was used to collect participants’ background information, including age, gender, educational level, and marital status. In addition, the questionnaire included items assessing the presence or absence of social support from different sources, namely siblings, children, grandchildren, sons and daughters-in-law, other relatives, friends, and neighbours. Information was also collected on the frequency of visits, satisfaction with the support received, length of institutionalisation, the presence or absence of chronic illnesses, and limitations in activities of daily living (ADL) (feeding, dressing/undressing, personal hygiene, using the toilet, and mobility). Information ADL was collected solely for descriptive purposes to characterise the functional profile of the sample and was not included in the inferential analyses.

#### 2.4.2. Loneliness

Loneliness was assessed using the Portuguese version of the UCLA Loneliness Scale (α = 0.905). The scale comprises 16 items across two dimensions: social isolation and affinities. Each item is rated on a 4-point Likert scale (1 = never, 4 = always), producing a total score ranging from 16 to 64. Higher scores indicate greater perceived loneliness, with a total score above 32 reflecting the presence of significant negative feelings of loneliness [40].

#### 2.4.3. Sleep Quality

Sleep quality was assessed using the Portuguese version of the Pittsburgh Sleep Quality Index (PSQI-PT), a standardised self-report measure of sleep quality over the previous month (α = 0.70). The instrument comprises 24 items, of which 19 contribute to the global score, and evaluates seven components: subjective sleep quality, sleep latency, sleep duration, habitual sleep efficiency, sleep disturbances, use of sleep medication, and daytime dysfunction. Each component is scored from 0 to 3, yielding a global score ranging from 0 to 21, with higher scores indicating poorer sleep quality. A global score >5 indicates significant sleep disturbance [41].

#### 2.4.4. Cognition

Cognition was assessed using the Portuguese version of the Montreal Cognitive Assessment (MoCA) [42] (α = 0.94). This instrument consists of 11 items spanning eight cognitive domains: attention, concentration, memory, working memory, executive function, language, visuoconstructional skills, and temporal and spatial orientation. Each item is scored dichotomously (0 or 1) based on the participant’s response, with a score of 1 assigned to correct answers. The total score ranges from 0 to 30, with higher scores indicating better cognitive function.

### 2.5. Data Collection

For this study, research teams were assembled to conduct the data collection process. To ensure procedural consistency and minimise potential sources of bias, all data collectors underwent prior training and received standardised instructions regarding the administration of the instruments.

**Figure 1:**
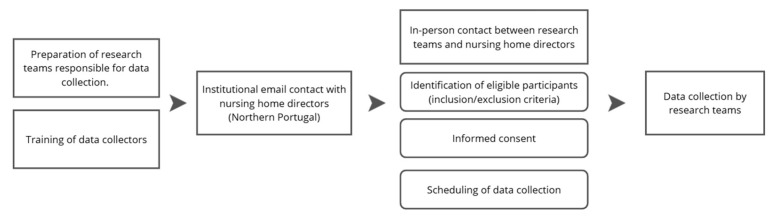
Procedure before data collection.

Data collection was carried out in accordance with agreements previously established between the directors of each participating nursing home and the research teams. A detailed overview of the procedures implemented before data collection is presented in Figure 1.

Given the wide range of measures required, a comprehensive data collection tool was developed using the EUSurvey platform. This tool was selected due to its efficiency in data entry and management, reduced administrative costs, and its capacity to minimise invalid responses and missing data [43].

Data collection proceeded in sequential phases to ensure that participants’ health status and availability were respected. Participants required approximately 60 min to complete the full set of instruments. During this period, members of the research teams remained available to provide support whenever necessary. Each set of instruments was assigned a unique alphanumeric code in order to preserve participant anonymity while enabling the documentation of the sequence of data collection within each participating nursing home.

The original data collection protocol required partial adaptation, as several nursing homes lacked a stable internet connection. In these settings, printed versions of the instruments were administered with the support of data collectors, and responses were subsequently entered into the electronic database. Thus, data quality was also closely monitored by data collectors in order to prevent missing data or prevent incorrect responses.

**Figure 2:**
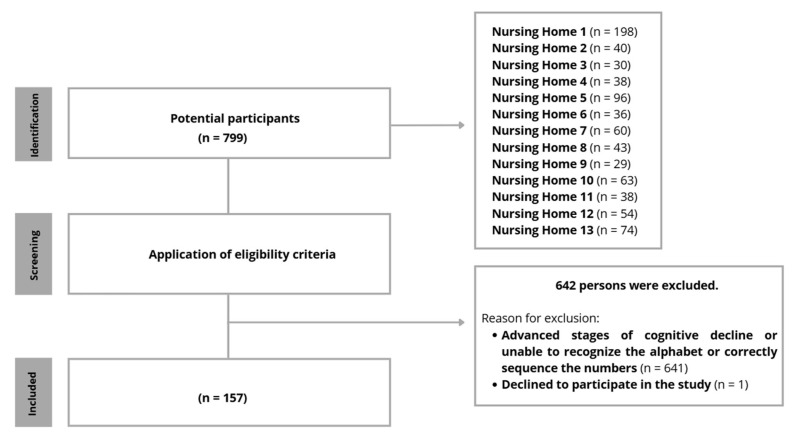
Participants recruitment and selection process.

Additional challenges emerged during the recruitment and assessment processes. A notable discrepancy was observed between the initially identified eligible population and the final study sample following the application of the predefined inclusion criteria. Many older adults presented advanced stages of cognitive decline and were unable to recognise the alphabet or correctly sequence the numbers, which resulted in their exclusion, as these abilities were required to complete mental health assessment instruments used in the study (Figure 2). Furthermore, although some residents met the eligibility criteria, a number declined to participate in the study, while others chose not to respond to specific items, particularly those concerning financial information.

### 2.6. Statistical Analysis

Data were analysed using the IBM Statistical Package for the Social Sciences (SPSS, version 30.0; IBM Corporation, Armonk, NY, USA). Prior to the analyses, the dataset was screened for missing values and potential outliers. Analyses were conducted using available cases for each variable. The distribution of variables was examined to assess normality and verify the assumptions required for parametric statistical analyses [44,45].

An a priori power analysis was conducted using G*Power 3.1 to determine the required sample size for the hierarchical multiple regression analysis (three steps, six predictors). Assuming a medium effect size (f^2^ = 0.15), an alpha level of 0.05, and a statistical power of 0.80, the minimum required sample size was approximately 97 participants. The final sample size (*n* = 157) exceeded this threshold, indicating that the study was adequately powered to detect medium-sized effects in the regression model. This sample size was also deemed sufficient for the independent samples *t*-tests and Pearson correlation analyses, based on conventional power requirements for detecting medium effect sizes (d ≈ 0.50; r ≈ 0.30), ensuring adequate statistical power across all statistical procedures conducted.

Descriptive statistics were used to characterise the sample. Continuous variables were summarised using means and standard deviations (SD), while categorical variables were presented as frequencies and percentages.

Pearson’s correlation coefficients were calculated to examine the relationships between age, loneliness dimensions, and sleep-related variables. Values above 0.80 indicated a very strong correlation, but values between 0.60 and 0.80 revealed a strong correlation, values between 0.40 and 0.60 indicated a moderate correlation, values between 0.20 and 0.40 indicated a weak correlation, and values below 0.20 were considered negligible [46]. To identify factors associated with sleep quality, a hierarchical multiple regression analysis was conducted with sleep quality as the dependent variable. Predictors were entered in successive steps based on theoretical considerations. In the first model, sociodemographic and institutional variables (age, frequency of visits, and time of institutionalisation) were included as baseline covariates. In the second model, perceived satisfaction with the social support network was added to assess its incremental contribution to the explained variance. In the final model, the loneliness dimensions (social isolation and affinities) were introduced as proximal psychosocial predictors, allowing the examination of their additional explanatory value beyond the variables included in the previous steps. Regression diagnostics were conducted to evaluate the assumptions of the multiple regression model. The distribution of standardised residuals and predicted values was examined to identify potential outliers and assess model adequacy. The standardised residuals ranged from −2.41 to 2.14, remaining within the acceptable range (±3), indicating the absence of significant outliers. The residuals were centred around zero, suggesting that the model errors were randomly distributed. These results indicate that the assumptions of linear regression were adequately met. The Durbin–Watson statistic was 1.899, indicating no evidence of autocorrelation in the residuals. Collinearity diagnostics were performed to assess potential multicollinearity among the independent variables. Condition indices and variance proportions were examined. Although moderate shared variance was observed between the loneliness dimensions (social isolation and affinities), no evidence of severe multicollinearity was detected. The condition indices remained within acceptable limits and did not indicate problematic collinearity among the predictors included in the regression models [47].

Statistical significance was set at *p* < 0.05 for all analyses.

### 2.7. Ethical Considerations

This study was conducted in accordance with the principles of the Declaration of Helsinki and received approval from the Ethics Committee of a major public university in Northern Portugal (CEICVS 007/2025). Before participation, all individuals were personally informed about the study procedures and provided written informed consent.

## 3. Results

### 3.1. Demographic Characteristics

Table 1 presents the results of the sample characterisation. The sample consisted of 157 institutionalised older adults. The majority of participants were female (72.6%), and the mean age was 83.3 years (SD = 7.24, range 65–97). Regarding marital status, most participants were widowed (55.4%), followed by single (19.7%), married (16.6%), and divorced individuals (8.3%).

In terms of educational level, nearly half of the sample had up to 4 years of schooling (49%). Additionally, 19.7% had attended school without completing any formal level of education, and 7.6% had no formal education. Smaller proportions had between 5 and 9 years of schooling (11.3%), secondary education (2.5%), or higher education (9.5%).

With respect to length of institutionalisation, the participants had been institutionalised for 1 to 5 years (49.7%) was the most frequent response, while 26.1% had been institutionalised for less than one year. About one-fifth had been institutionalised for 6 to 10 years (18.5%), and a small proportion for 11 to 15 years (3.8%) or 16 years or more (1.9%).

Almost all participants reported the presence of chronic diseases (98.7%). Furthermore, 75.2% of the sample presented limitations in activities of daily living. The most frequent limitations were related to personal hygiene (63.1%), walking (58%), and dressing (49%). Limitations in transferring were reported by 27.4% of participants, while fewer participants experienced limitations in toilet use (21%) and feeding (1.9%).

### 3.2. Relation Between Sex, Loneliness and Sleep

Independent samples *t*-tests were conducted to examine potential sex differences in social support, sleep variables, and loneliness. The results indicated no significant differences between women and men across all variables assessed (Table 2).

Specifically, social support satisfaction did not differ between women (M = 9.26, SD = 1.93) and men (M = 9.35, SD = 1.68), t(155) = −0.257, *p* = 0.798, Cohen’s d = −0.046. Similarly, no significant sex differences were observed for PSQI sleep latency (women: M = 1.57, SD = 1.11; men: M = 1.44, SD = 1.26; t(155) = 0.624, *p* = 0.533, d = 0.112), PSQI sleep duration (women: M = 0.35, SD = 0.72; men: M = 0.50, SD = 0.58; t(112) = −1.014, *p* = 0.313, d = −0.221), PSQI sleep efficiency, PSQI sleep disturbance, PSQI use of sleep medication, PSQI daytime dysfunction, PSQI global sleep quality (women: M = 9.43, SD = 4.22; men: M = 8.70, SD = 4.18; t(155) = 0.972, *p* = 0.332, d = 0.174), UCLA total score (women: M = 46.5, SD = 14; men: M = 46.4, SD = 14.4; t(155) = 0.004, *p* = 0 0.997, d = 0.001), UCLA social isolation (women: M = 31.7, SD = 9.54; men: M = 32.1, SD = 9.98; t(155) = −0.194, *p* = 0.846, d = -0.035), or UCLA affinities (women: M = 14.8, SD = 4.96; men: M = 14.5, SD = 4.95; t(155) = 0.389, *p* = 0.658, d = 0.070).

### 3.3. Correlation Analysis

A Person correlation analysis was conducted to explore the relationships between age, loneliness (UCLA total score and its dimensions), and sleep-related variables (Table 3). Higher PSQI scores indicate poorer sleep outcomes (greater sleep impairment).

Age showed a small but significant positive correlation with the UCLA total score (r = 0.231, *p* < 0.01), as well as with social isolation (r = 0.239, *p* < 0.01) and affinities (r = 0.189, *p* < 0.05), suggesting that older participants tended to report higher loneliness levels.

Regarding sleep variables, the UCLA total score was positively correlated with the PSQI sleep efficiency component (r = 0.273, *p* < 0.01), daytime dysfunction (r = 0.211, *p* < 0.01), and overall sleep quality (r = 0.588, *p* < 0.01), indicating that higher loneliness was associated with poorer sleep outcomes. Similar patterns were observed for the loneliness dimensions. Social isolation showed positive correlations with sleep efficiency (r = 0.262, *p* < 0.01), daytime dysfunction (r = 0.202, *p* < 0.05), and sleep quality (r = 0.573, *p* < 0.01). Likewise, affinities were also positively associated with poorer sleep efficiency (r = 0.264, *p* < 0.01), daytime dysfunction (r = 0.207, *p* < 0.01), and global sleep quality (r = 0.552, *p* < 0.01).

Significant associations were also observed between PSQI components. Subjective sleep quality was negatively correlated with sleep efficiency (r = −0.211, *p* < 0.05), sleep latency (r = −0.313, *p* < 0.01), sleep duration (r = −0.295, *p* < 0.01), habitual sleep efficiency (r = −0.252, *p* < 0.01), sleep disturbance (r = −0.220, *p* < 0.05) and daytime dysfunction (r = −0.209, *p* < 0.05). This pattern reflects the PSQI scoring structure, in which higher scores represent poorer outcomes across components.

The PSQI sleep efficiency showed a strong positive correlation with sleep duration (r = 0.590, *p* < 0.01), and moderate positive correlations with habitual sleep efficiency (r = 0.267, *p* < 0.01), sleep disturbance (r = 0.183, *p* < 0.05), daytime dysfunction (r = 0.189, *p* < 0.05), and global sleep quality (r = 0.373, *p* < 0.01).

Global sleep quality demonstrated significant positive correlations with several PSQI components, including sleep latency (r = 0.199, *p* < 0.05), sleep duration (r = 0.326, *p* < 0.01), habitual sleep efficiency (r = 0.447, *p* < 0.01), sleep disturbance (r = 0.326, *p* < 0.01), use of sleep medication (r = 0.541, *p* < 0.01), and daytime dysfunction (r = 0.565, *p* < 0.01), indicating that poorer sleep quality was consistently associated with greater impairment across these domains.

Additionally, sleep time was positively correlated with habitual sleep efficiency (r = 0.583, *p* < 0.01) and daytime dysfunction (r = 0.159, *p* < 0.05), and negatively correlated with sleep latency (r = −0.198, *p* < 0.05) and sleep duration (r = −0.204, *p* < 0.05).

### 3.4. Predictors of Sleep Quality

A hierarchical multiple regression analysis was performed to identify predictors of global sleep quality (PSQI total score), with higher scores indicating poor sleep quality (Table 4). Sociodemographic variables were entered in the first block (age, frequency of visits, and length of institutionalisation). This model was statistically significant (F(3,153) = 3.11, *p* = 0.028), explaining 5.7% of the variance in sleep quality (R^2^ = 0.057; adjusted R^2^ = 0.039). Within this model, age emerged as a significant predictor of poorer sleep quality (β = 0.212, *p* < 0.01), while frequency of visits and time of institutionalisation were not significant predictors.

In the second model, satisfaction with the social support network was added. This step did not significantly improve the model, and satisfaction with social support was not significantly associated with PSQI global sleep quality (β = −0.086, *p* = 0.292). The model remained statistically significant (F(4,152) = 2.61, *p* = 0.038), explaining 6.4% of the variance in sleep quality (R^2^ = 0.064; adjusted R^2^ = 0.040).

In the final model, UCLA dimensions (social isolation and affinities) were introduced. The full model was statistically significant (F(6,150) = 15.36, *p* < 0.001), explaining 38.1% of the variance in sleep quality (R^2^ = 0.381; adjusted R^2^ = 0.356). In this model, frequency of visits became a significant predictor (β = −0.166, *p* < 0.05) indicating the more frequent visits were associated with better sleep quality (lower PSQI scores). Social isolation emerged as the strongest predictor of poor sleep quality (β = 0.393, *p* < 0.01). Affinities showed a positive association with sleep quality but did not reach statistical significance (β = 0.213, *p* = 0.072). Age and time of institutionalisation were no longer predictors in the final model.

## 4. Discussion

A large number of potential participants were excluded from the study due to dementia screening, reflecting the high prevalence of dementia among the older adults living in nursing homes. This is consistent with the substantial proportion of older adults with cognitive impairment living in institutional settings. The overrepresentation of individuals with dementia in nursing homes is closely linked to the limited availability of community-based care options. In many cases, the lack of formal home care services, insufficient support for informal caregivers, and the absence of alternative living arrangements such as assisted living facilities contribute to the need for institutionalisation. As a result, nursing homes often become the primary response for individuals with complex care needs and cognitive decline, highlighting gaps in community care provision.

The findings show that older adults aged 65 years and over residing in nursing homes in Northern Portugal are predominantly aged 80 years or older, female, widowed, and characterised by low levels of formal education. This sociodemographic profile reflects demographic trends observed among older adults living in nursing homes across Europe [48]. Moreover, our results point to a pronounced overrepresentation of women within the Portuguese context, a pattern also identified in a study conducted in Southern Portugal [49]. Taken together, these findings suggest that the proportion of institutionalised older women in Portugal may be higher than reported in other European countries. This may partly reflect their lower likelihood of ageing in place [50], combined with their longer life expectancy, which increases the chances of surviving into advanced ages with functional limitations requiring long-term care.

Nevertheless, some differences emerge when comparing our findings with previous research. Our sample presents a substantially higher prevalence of chronic diseases (98.7% vs. 77%) [48]. Thus, although the severity of limitation in activities of daily living was not differentiated, 75.2% of participants reported some level of limitation, a proportion higher than previously reported (65.1%) [48]. Mobility-related difficulties were particularly prominent, with 58% of participants having limitations in walking. Similar patterns have been observed among older adults living in nursing homes across Europe, where approximately 40% of individuals report the use of mobility aids [48]. In Portugal, admission to nursing homes frequently occurs at relatively advanced stages of dependency, particularly when health and functional needs exceed what can be managed at home, especially in the absence of informal caregiving [51]. The later institutionalisation may help explain the high levels of chronic conditions and functional limitations observed in the present study. Additionally, this difference may reflect the high prevalence of multimorbidity in Northern Portugal [52], as well as potential health consequences emerging in the post-pandemic period [53].

Consistent with recent literature, our findings revealed no significant sex differences in social support, loneliness, or sleep quality. Previous studies using logistic regression models initially suggested that men experienced better sleep than women; however, these differences became non-significant once common age-related factors, including depression and cognitive decline, were considered. These results indicate that sleep quality among older adults living in nursing homes is influenced by a broad set of determinants beyond sex, reflecting underlying health conditions such as cognitive function and mood [54]. Likewise, another study conducted within this setting found no statistically significant sex differences in loneliness, although women tended to report slightly higher levels than men [55]. Overall, sex alone does not seem to account for differences in sleep or loneliness in this population. Instead, these outcomes appear to arise from the multifactorial interplay of health-related and psychosocial factors.

The present study revealed significant associations between age, loneliness, and sleep-related variables in older adults living in nursing homes. Age was positively, although modestly, correlated with the total UCLA loneliness score (r = 0.231, *p* < 0.01) and its dimensions of social isolation (r = 0.239, *p* < 0.01) and affinities (r = 0.189, *p* < 0.05), indicating that older participants tend to report slightly higher levels of perceived loneliness. This finding aligns with previous research suggesting that advancing age is a risk factor for loneliness, due to reductions in social networks and life transitions such as bereavement and retirement [36,56].

Regarding sleep, the total UCLA score and its dimensions were positively associated with sleep efficiency, daytime dysfunction, and overall perceived sleep quality. These associations are consistent with meta-analytic evidence indicating that loneliness is significantly associated with poor sleep quality in older adults, with lonely individuals more likely to experience sleep disturbances [35]. Similarly, Yu et al. [57] found that both loneliness and social isolation were linked to poorer sleep quality when measured objectively and subjectively in a national cohort of older adults. These studies reinforce the robustness of the relationship between social isolation, loneliness, and sleep in later life, and support our findings in an institutionalised sample. Interestingly, the positive correlations between loneliness and sleep efficiency may appear counterintuitive but are compatible with prior observations that subjective and objective sleep measures do not always converge in older adults [57]. Participants who are socially isolated may have fewer external disturbances, leading to seemingly consolidated nocturnal sleep, while simultaneously experiencing greater daytime dysfunction and lower perceived sleep quality, suggesting that the subjective experience of sleep is influenced by psychosocial factors beyond sleep continuity.

Regarding subjective sleep quality and sleep medication use, our findings are in line with previous literature, suggesting that poorer subjective sleep quality is associated with an increased likelihood of sleep medication use, although this association is likely influenced by additional factors [58]. Moreover, total sleep time was positively correlated with habitual sleep efficiency and daytime dysfunction, and negatively correlated with sleep latency and sleep duration anomalies. This pattern reflects the complex interplay between sleep timing, continuity, and perceived sleep quality, which has been documented in longitudinal studies showing bidirectional relationships between poor sleep and increasing loneliness over time [59]. These findings highlight that interventions targeting sleep improvement may also mitigate loneliness-related daytime dysfunction, and vice versa. Taken together, these results underscore the importance of integrated approaches in nursing homes that simultaneously address social engagement and sleep health.

Programmes promoting meaningful social interactions, enhancing interpersonal connectedness, and providing structured daytime activities may reduce perceived loneliness, while interventions targeting sleep hygiene, environmental conditions, and daily routines may improve both sleep efficiency and daytime functioning [35,56]. Moreover, there are several variables that contribute to sleep disturbances, and these cannot be excluded from the discussion, including hypervigilance, reduced physical activity, and decreased cognitive engagement [35,56].

Overall, our findings corroborate and extend existing literature showing that loneliness and social isolation are significantly linked to subjective sleep quality and daytime functioning in older adults. These results reinforce the need for integrated care approaches that consider social, psychological, and physiological factors to enhance the overall quality of life of older adults in nursing homes [36,56,57,58,59].

In the present study, we also examined predictors of sleep quality among older adults living in nursing homes. The results reveal a progressive shift in explanatory factors across the regression models, whereas sociodemographic variables accounted for only a limited proportion of the variance in sleep quality, the inclusion of psychosocial dimensions markedly enhanced the model’s explanatory power.

In the first model, age emerged as the only significant predictor of sleep quality. This finding can be interpreted in light of age-related changes in sleep architecture. With advancing age, sleep is characterised by reduced deep sleep, increased fragmentation, more frequent nocturnal awakenings, and greater difficulty maintaining sleep [35,60]. Consistent with our findings, evidence from community-dwelling older adults has also identified age as a predictor of poorer sleep quality [61]. In our study, despite its statistical significance, age explained only a modest proportion of the variance in sleep quality, suggesting that biological ageing alone provides a limited explanation for interindividual differences in sleep quality. This finding highlights the relevance of considering additional psychosocial factors when examining sleep quality in later life. Interestingly, the length of institutionalisation and frequency of visits were not significant predictors in the initial model. This finding suggests that these variables may not directly determine sleep quality when considered independently. Their influence may operate indirectly, through psychosocial pathways, by shaping residents’ emotional well-being and sense of social connectedness.

In the second model, satisfaction with the social support network did not significantly improve the variance within sleep quality, and age remained the only significant predictor. Although satisfaction with the social support network reflects a personal evaluation of perceived support, it may represent a global assessment that fails to capture more specific relational experiences, such as meaningful interactions or feelings of social disconnection. This distinction is widely recognised in the literature, as perceived social support, loneliness, and social isolation represent related but conceptually distinct constructs. A recent meta-synthesis exploring older adults’ experiences of loneliness in nursing homes found that loneliness is a subjective and multifaceted experience shaped by personal loss, environmental transitions, and social disengagement. Residents described loneliness as painful and difficult to cope with, reflecting a complex interaction between internal perceptions and external circumstances rather than the lack of social support. Furthermore, the authors found that older adults living in this setting may maintain social contacts while still experiencing loneliness [62], which highlights that objective indicators may inadequately demonstrate the emotional quality of interpersonal relationships in this setting. Accordingly, the absence of a significant predictive effect of satisfaction with social support may reflect the limited ability of this measure to fully represent the subjective experience of loneliness among older adults living in nursing homes.

The inclusion of loneliness dimensions in Model 3 substantially increased the explanatory capacity of the regression model, with the explained variance reaching 38.1%. This represents a notable improvement over previous models and suggests that loneliness is a particularly relevant psychosocial factor associated with sleep quality in this sample. The magnitude of this increase indicates that subjective social experiences may play a more prominent role in explaining sleep variability than sociodemographic characteristics alone. Loneliness is generally defined as the subjective perception of insufficient or unsatisfactory social relationships, reflecting a discrepancy between desired and actual social connections. Unlike objective social isolation, loneliness captures the emotional quality of social relationships and therefore represents a psychologically meaningful experience that may influence multiple aspects of well-being. In the context of ageing, this phenomenon becomes particularly relevant, as life transitions such as retirement, bereavement, or reduced mobility may alter social networks and increase vulnerability to perceived social disconnection.

Several mechanisms may explain the relationship between loneliness and sleep disturbances. Loneliness is frequently associated with increased rumination, worry, and negative cognitive appraisal, processes that may elevate cognitive arousal during the pre-sleep period and interfere with the initiation and maintenance of sleep. In addition, feelings of social disconnection may contribute to a state of heightened psychological alertness, potentially reflecting perceived vulnerability in the absence of supportive social relationships. Such processes may hinder the relaxation and psychological disengagement required for restorative sleep. Recent empirical research provides growing support for the association between loneliness and sleep quality. A systematic review and meta-analysis conducted by Deng et al. [35], including more than 23,000 participants, found that older adults experiencing loneliness were significantly more likely to report poor sleep quality compared with those reporting lower levels of loneliness. These findings highlight the robustness of the association across diverse populations and methodological approaches.

Similarly, longitudinal evidence suggests that loneliness may contribute to the development of sleep problems over time. For example, Yu et al. [63] reported that loneliness was significantly associated with the onset of insomnia symptoms among middle-aged and older adults, even after controlling for sociodemographic characteristics and health-related variables. This finding suggests that loneliness may operate as a risk factor for sleep disturbances rather than merely a correlate of poor sleep. Further evidence indicates that loneliness may also function as an important psychosocial pathway linking social relationships to sleep outcomes. Using longitudinal data, Jiang et al. [38] demonstrated that loneliness mediated the relationship between social isolation and sleep problems among older adults, highlighting the importance of subjective social experiences in understanding sleep health. In a similar perspective, Seo et al. [56] found that loneliness played a mediating role in the relationship between social relationships and sleep quality, suggesting that the emotional dimension of social connection may be particularly relevant for sleep outcomes.

The findings of the present study are consistent with this growing body of literature, indicating that loneliness represents a meaningful psychosocial determinant of sleep quality in later life. Importantly, the substantial increase in explained variance observed after the inclusion of loneliness variables suggests that psychosocial factors may account for a considerably greater proportion of variability in sleep outcomes than sociodemographic characteristics alone. While demographic variables provide important contextual information, they may not adequately capture the emotional and relational experiences that shape sleep health. Moreover, recent research highlights the broader social context in which sleep health develops. For instance, Wang et al. [64] demonstrated that loneliness and sleep quality may act as interconnected mechanisms linking social participation to health outcomes in older adults. Such findings reinforce the idea that sleep should be understood not only as a biological process but also as a phenomenon embedded within social and relational contexts. From a practical perspective, these findings suggest that interventions aimed at improving sleep among older adults may benefit from addressing loneliness and promoting social connectedness. Strategies designed to enhance social engagement, strengthen interpersonal relationships, and increase opportunities for meaningful social participation may contribute not only to improved psychological well-being but also to better sleep outcomes.

Despite the evidence presented in this section, the relevance and impact of environmental factors should not be overlooked. Noise, air quality and humidity, and room cohabitation have been identified as key determinants of sleep quality and may influence sleep outcomes through indirect pathways, ultimately impacting sleep quality and sleep continuity [65].

Finally, future research should further investigate the mechanisms underlying the relationship between loneliness and sleep, particularly through longitudinal and multidisciplinary approaches. Exploring how social environments, emotional well-being, and behavioural factors interact to influence sleep may provide valuable insights for the development of interventions targeting both loneliness and sleep health in ageing populations. Overall, the results reinforce the growing body of evidence suggesting that loneliness represents a significant psychosocial determinant of sleep quality and may constitute an important risk factor for sleep disturbances in later life.

### Limitations

Although this study provides new insights into sleep quality and loneliness among older adults living in nursing homes, several limitations should be acknowledged. First, despite the inclusion of multiple institutions (*n* = 13) and a large pool of potential participants (*n* = 799), only a small proportion (approximately 20%) met the inclusion criteria. This may indicate a selection bias and limits the representativeness of residents with more severe cognitive decline, who were not included. It also reflects the high levels of physical and/or cognitive dependence commonly observed in this population, which constrained broader participation.

Second, the cross-sectional design does not allow causal inferences, restricting interpretation to associations at a single point in time. In addition, the use of self-report measures may introduce response and information bias, particularly given the inclusion of participants with mild cognitive impairment, despite prior cognitive screening using the MoCA.

Third, relevant clinical and nursing-related variables, such as depression, pain, psychotropic medication use, sleep medication use (type, dosage, duration) and functional status, were not assessed and may have influenced the observed outcomes. Moreover, institutional factors (e.g., routines, staffing, environmental conditions, and social dynamics) may have also affected sleep and loneliness, limiting the generalisability of the findings across different care settings.

Future longitudinal studies are recommended to clarify the directionality of these associations and to inform nursing interventions targeting social integration and sleep quality in institutionalised older adults [58].

## 5. Conclusions

This finding suggests that loneliness represents a key psychosocial determinant of sleep quality among older adults living in nursing homes, exceeding the influence of traditionally recognised individual characteristics.

Loneliness appears to be an important psychosocial correlate of sleep quality among older adults within this setting, showing a stronger association with sleep outcomes than sociodemographic factors. While age may modestly contribute to sleep disruption, perceived social disconnection is more closely associated with poorer sleep quality and greater daytime dysfunction. These findings underscore the need for integrated interventions in nursing homes that address both social engagement and sleep health. Future longitudinal studies are needed to clarify causal relationships and inform targeted interventions. Given the cross-sectional design, these results should be interpreted as associations rather than causal relationships. Nevertheless, they suggest that both social engagement and sleep health should be addressed in an integrated manner within nursing home care. The findings highlight the need for a systematic and integrated approach to assessing loneliness and sleep quality in institutional settings.

From a nursing practice perspective, these results highlight the need to integrate the assessment of loneliness into the clinical monitoring of older adults in institutional settings. personalised nursing interventions that promote social engagement, emotional support, and the development of meaningful relationships may play a key role in improving sleep quality.

The results also underscore the importance of integrated institutional policies that consider sleep and loneliness as interdependent dimensions of care, including regular screening, personalised care plans, and programmes promoting social participation. Such measures may significantly inform social and health professionals to contribute for good practice and to dignified care and the well-being of older adults living in nursing homes.

## Figures and Tables

**Table 1:** Demographic characteristics of respondents.

	*n* (%)	M (SD)
Gender	157	
Female	114 (72.6)	
Male	43 (27.4)	
Age	157	83.3 (7.24)
Marital status	137	
Single	31 (19.7)	
Married	26 (16.6)	
Windowed	87 (55.4)	
Divorced	13 (8.3)	
Educational level	157	
No formal education	12 (7.6)	
No completed level of education	31 (19.7)	
Up to 4 years of schooling	77 (49)	
Between 5 and 9 years of schooling	18 (11.3)	
Secondary education	4 (2.5)	
Higher education	15 (9.5)	
Time since institutionalisation	157	
<1 year	41 (26.1)	
1–5 years	78 (49.7)	
6–10 years	29 (18.5)	
11–15 years	6 (3.8)	
≥16 years	3 (1.9)	
Chronical diseases	157	
Yes	155 (98.7)	
Limitations in activities of daily living	118 (75.2)	
Limitations in dressing	77 (49)	
Limitations in hygiene	99 (63.1)	
Limitations in feeding	3 (1.9)	
Limitations in toilet use	33 (21)	
Limitations in walking	91 (58)	
Limitations in transferring	43 (27.4)	

**Table 2:** Sex differences in loneliness, social support and sleep quality.

	Women		Men				
	N	M (SD)	N	M (SD)	t	*p*	d
Social support satisfaction	114	9.26 (1.93)	43	9.35 (1.68)	−0.257	0.798	−0.046
PSQI sleep latency	114	1.57 (1.105)	43	1.44 (1.26)	0.624	0.533	0.112
PSQI sleep duration	86	0.35 (0.716)	28	0.50 (0.577)	−1.014	0.313	−0.221
PSQI sleep efficiency	86	2.63 (0.720)	28	2.71 (0.810)	−0.534	0.594	−0.116
PSQI sleep disturbance	114	1.28 (0.541)	43	1.23 (0.427)	0.583	0.561	0.094
PSQI use sleep medication	86	2.24 (1.30)	28	2.36 (1.25)	−0.403	0.688	−0.088
PSQI daytime dysfunction	114	1.20 (1.10)	43	1.16 (1.17)	0.194	0.846	0.035
PSQI global sleep quality	114	9.43 (4.22)	43	8.70 (4.18)	0.972	0.332	0.174
UCLA total score	114	46.5 (14)	43	46.4 (14.4)	0.004	0.997	0.001
UCLA Social isolation	114	31.7 (9.54)	43	32.1 (9.98)	−0.194	0.846	−0.035
UCLA Affinities	114	14.8 (4.96)	43	14.5 (4.95)	0.389	0.658	0.070

Note. Sample sizes vary across variables due to missing responses. Valid data were available for most variables for *n* = 157. However, PSQI sleep duration, habitual sleep efficiency, and use of sleep medication were calculated only for participants who provided complete responses to the relevant PSQI items (*n* = 114).

**Table 3:** Pearson Correlations (*n* = 114).

	1	2	3	4	5	6	7	8	9	10	11	12	13	14
1. Age	1													
2. UCLA total score	0.231 **	1												
3. Social isolation	0.239 **	0.981 **	1											
4. Affinities	0.189 *	0.926 **	0.836 **	1										
5. PSQI subjective sleep quality	−0.052	0.145	−0.139	0.126	1									
6. PSQI sleep efficiency	−0.062	0.273 **	0.262 **	0.264 **	−0.211 *	1								
7. PSQI sleep latency	0.053	−0.160 *	−0.173 *	−0.115	−0.313 **	0.104	1							
8. PSQI sleep duration	−0.080	0.030	0.044	−0.004	−0.295 **	0.590 **	0.175	1						
9. PSQI habitual sleep efficiency	0.164	0.069	0.050	0.093	−0.252 **	0.267 **	−0.004	0.165	1					
10. PSQI sleep disturbance	0.008	0.082	0.067	0.101	−0.220 *	0.183 *	0.039	0.187 *	0.214 *	1				
11. PSQI use of sleep medication	0.010	−0.110	−0.107	−0.095	0.214 *	−0.031	−0.039	−0.161	0.008	−0.007	1			
12. PSQI daytime dysfunction	0.131	0.211 **	0.202 *	0.207 **	−0.209 *	0.189 *	0.055	0.121	0.323 **	0.258 **	0.196 *	1		
13. Global sleep quality (PSQI total score)	0.197 *	0.588 **	0.573 **	0.552 **	−0.116	0.373 **	0.199 *	0.326 **	0.447 **	0.326 **	0.541 **	0.565 **	1	
14. Sleep time	0.106	0.065	0.068	0.052	−0.013	−0.045	−0.198 *	−0.204 *	0.583 **	0.087	−0.026	0.159 *	0.078	1

** *p* < 0.01; * *p* < 0.05. Note: Positive correlations between loneliness and PSQI sleep variables indicate poorer sleep outcomes, since higher PSQI scores reflect greater impairment.

**Table 4:** Hierarchical multiple regression analysis predicting PSQI global sleep quality (PSQI total score).

	Model 1 (*n* = 156)					Model 2 (*n* = 156)					Model 3 (*n* = 156)				
	B	SE B	β	t	*p*	B	SE B	β	t	*p*	B	SE B	β	t	*p*
Age	0.123	0.046	0.212	2.678	0.008	0.133	0.047	0.230	2.842	0.005	0.053	0.040	0.091	1.341	0.182
Frequency of visits	−0.361	0.222	−0.130	−1.627	0.106	−0.324	0.224	−0.117	−1.446	0.150	−0.459	0.185	−0.166	−2.477	0.014
Time of institutionalisation	0.143	0.380	0.029	0.369	0.712	0.121	0.381	0.025	0.318	0.751	−0.158	0.314	−0.033	−0.505	0.615
Social support satisfaction						−0.196	0.185	−0.086	−1.058	0.292	−0.111	0.152	−0.049	−0.732	0.465
Social isolation											0.172	0.052	0.393	3.282	0.001
Affinities											0.182	0.100	0.213	1.814	0.072
R^2^ (R^2^ adju)	0.057 (0.039)					0.064 (0.040)					0.381 (0.356)				
ΔR^2^	0.057					0.007					0.317				
F for change in R^2^	3.109				0.028	2.614				0.038	15.361				<0.001

Note: Dependent variable: PSQI total score (higher scores indicate poorer sleep quality). B = unstandardised coefficient; SE = standard error; β = standardised coefficient. Model 1 predictors: Age, Frequency of visits, Time of institutionalisation. Model 2 adds: Social support satisfaction. Model 3 adds: Social isolation, Affinities.

## Data Availability

The dataset is available on reasonable request from the corresponding author due to ethical restrictions.

## Public Involvement Statement

No public involvement in any aspect of this research.

## Guidelines and Standards Statement

This manuscript was prepared in accordance with the STROBE Statement (Strengthening the Reporting of Observational Studies in Epidemiology) for reporting cross-sectional observational studies.
